# C-reactive protein to high-density lipoprotein cholesterol ratio: an independent risk factor for diabetic retinopathy in type 2 diabetes patients

**DOI:** 10.3389/fnut.2025.1537707

**Published:** 2025-03-04

**Authors:** Yuan Zhang, Guanhua Chen, Weimin Wang, Yali Jing

**Affiliations:** ^1^Department of Endocrinology, Endocrine and Metabolic Disease Medical Center, Nanjing Drum Tower Hospital Clinical College of Nanjing University of Chinese Medicine, Nanjing, China; ^2^Branch of National Clinical Research Centre for Metabolic Diseases, Nanjing, China; ^3^Department of Endocrinology, Endocrine and Metabolic Disease Medical Center, Nanjing Drum Tower Hospital, Affiliated Hospital of Medical School, Nanjing University, Nanjing, China

**Keywords:** diabetic retinopathy (DR), the CRP to HDL-C ratio, biomarker, type 2 diabetes mellitus (T2DM), dyslipidemia, inflammation

## Abstract

**Background and objective:**

Diabetic retinopathy (DR) is associated with abnormal lipid metabolism and inflammation. However, a single lipid or inflammatory parameter cannot accurately predict the prognosis of DR independently, because it is prone to be affected by various confounding factors. This study aimed to explore the relationship between the inflammation-lipid indicator C-reactive protein (CRP)/high-density lipoprotein cholesterol (HDL-C) and DR occurrence in subjects with type 2 diabetes mellitus (T2DM).

**Methods:**

This hospital-based retrospective study included 784 T2DM patients. Diabetic retinopathy was diagnosed by nonmydriatic fundus photography and/or fundus examination apparatus. T2DM patients were divided into non-DR and DR groups. Demographics variables, clinical history and serum biochemical indicators of the subjects were collected. We also calculated the CRP/HDL-C ratio. The association between the CRP/HDL-C and DR was assessed using multivariate logistic regression analyses.

**Results:**

A total of 784 participants, 612 without DR and 172 with DR, were included in the final sample analysis. Compared with non-DR participants, the DR diagnostic group had significantly higher CRP/HDL-C (4.03 ± 1.67 vs. 2.66 ± 0.97; *p* < 0.001). Then, the patients were grouped based on the quartiles of CRP/HDL-C, there was a gradual increase in the prevalence of DR was noted in T2DM patients along with the increased quartile of the CRP/HDL-C ratio (Q1: 7.65%; Q2: 15.31%; Q3: 19.90%; Q4: 44.90%; *p* = 0.028). After adjustment for the impact of various covariates, the odds ratio (OR) of the third and fourth vs. the first quartile of CRP/HDL-C were 2.905 (95% confidence interval [CI]: 1.372 ~ 6.152, *p* = 0.005) and 9.938 (95% CI: 4.987 ~ 19.804, *p* < 0.001), respectively. Further, multivariate logistic regression model showed that the CRP/HDL-C ratio (OR 3.176, 95% CI: 1.280 ~ 7.877, *p* = 0.013) was identified as risk factor for DR. Moreover, the area under the curve (AUC) to evaluate the predictive value of CRP/HDL-C for the risk of DR occurrence was 0.752 (95% CI: 0.711 ~ 0.794).

**Conclusion:**

The ratio of C-reactive protein (CRP) to high-density lipoprotein cholesterol (HDL-C) is associated with DR in patients with T2DM, and CRP/HDL-C may be an effective marker to help identify the risk of DR in patients with T2DM.

## Introduction

With demographic and lifestyle changes, the global prevalence of diabetes mellitus (DM) continues to increase. The global prevalence of diabetes in adults was estimated to be rising to 12.2% by 2045, of which 90% is type 2 diabetes mellitus (T2DM) (1). Diabetic retinopathy (DR), as one of the common microvascular complications of diabetes, jeopardizes the patient’s vision level and even leads to blindness, affecting patients’ survival and quality of life (2). The epidemiologic projections indicated that the prevalence of DR was 22.27% among people with DM, which increases the risk of life-threatening systemic vascular complications such as stroke, coronary heart disease and heart failure, and the global burden of DR was predicted to remain high until 2045 (3, 4). With the increase in the number of diabetic patients and the aging population, DR has become a great challenge to avoid visual impairment and blindness. However, because of the low compliance rate for DR screening, many patients with DR did not seek appropriate medical care until progressed to an irreversible stage. Thus, exploring simple and reliable biomarkers to predict the development of DR is important in comprehensive clinical management.

Previous studies have demonstrated that DR is associated with the duration of diabetes, poor glycemic control, dyslipidemia, hypertension, inflammatory response and nerve damage (5–8). In addition, several studies have reported that high-density lipoprotein cholesterol (HDL-C) and C-reactive protein (CRP) are risk factors for diabetes and cardiovascular disease (9, 10), however, a single lipid indicator or a single inflammatory indicator is susceptible to multiple confounding factors and may not accurately predict the risk of developing DR. The CRP/HDL-C as a composite indicator, is more capable of comprehensively assessing the correlation between the inflammatory system and lipid levels and the risk of DR. As an inflammation-lipid composite marker, the ratio of CRP to HDL-C has been shown to be associated with cardiovascular and metabolism-related diseases (11–13).

Therefore, in the present study, we sought to explore the relationship between the inflammation-lipid indicator CRP/HDL-C and DR occurrence in a cross-sectional study. We aimed to corroborate whether the CRP/HDL-C ratio is an independent predictor of DR occurrence. The findings of this study are anticipated to bridge the gap between DR and markers of inflammation-lipid function and have clinical implications for the management of DR.

## Materials and methods

### Study design and population

This study retrospectively collected patients with T2DM who were hospitalized in the Endocrinology Department of Nanjing Drum Tower Hospital between January 2015 and January 2019, aged between 18 and 80 years. T2DM was diagnosed according to the 2003 American Diabetes Association criteria (14). The International Clinical Diabetic Retinopathy Scale was used to group the presence or absence of retinopathy (15). Based on the presence or absence of diabetic retinopathy, the study subjects were divided into DR group and non-DR group. Subjects with combined acute complications of diabetes mellitus, severe infections, dysfunction of vital internal organs and tissues (heart, liver, kidney, etc.), and malignant tumors were excluded. Patients with hormonal or additive medications within the last 3 months that could have an effect on endocrine metabolism were also excluded.

### Data collection

We reviewed the electronic medical records of all patients in order to collect demographic and biochemical variables retrospectively. Information on each participant, including their age, systolic blood pressure (SBP), diastolic blood pressure (DBP), smoking, drinking, diabetes duration was collected using standard technical methods. Blood specimens of each individual were collected for laboratory analysis between 8:00 and 10:00 am after overnight fasting for at least 8 h. Glycated hemoglobin (HbA1c) concentrations were measured by high-performance liquid chromatography (HLC-73G8, Tosoh, Japan), fasting plasma glucose (FPG) and 2-h postprandial blood glucose (2 h-PG) were tested using a hexokinase method (TBA-200FR, Tokyo, Japan). The automatic biochemical analyzer (Kyowa Medex Co., Ltd., Tokyo, Japan) was used to detect concentrations of triglyceride (TG), total cholesterol (TC), HDL-C, low-density lipoprotein cholesterol (LDL-C), alanine aminotransferase (ALT), aspartate aminotransferase (AST), blood urea nitrogen (BUN), creatinine (Cr), uric acid (UA), following the manufacturer’s instructions. C-reactive protein was measured using the nephelometric method on the Siemens Dade Behring BN II analyzer (Siemens Healthcare Diagnostics, Deerfield, IL). We also calculated the CRP/HDL-C ratio. Additionally, it should be noted that some of the patients included in this study were on medications for cardiovascular diseases, which has been considered in our interpretation of the results.

### Statistical analysis

Continuous variables were represented as mean ± standard deviation or median (quartiles), and categorical variables were represented as frequencies or percentages. The Statistical differences in means and proportions between two independent groups were determined using independent sample t-test (normal distribution), Kruskal–Wallis test (skewed distribution), and Chi-square test (categorical variables). The binary logistic regression analysis was used to examine the overall DR risk at the CRP/HDL-C ratio quartile. Multivariate logistic regression analyses were used to determine the associations between the CRP/HDL-C ratio and incident DR, when the CRP/HDL-C ratio level was treated as a continuous variable. The results were evaluated within a 95% confidence interval (CI) and at a significance level of two-sided *p*-value less than 0.05. All data were examined by adopting the program SPSS Statistics software version 27.0 (IBM SPSS Inc., Chicago, USA). Statistics were considered significant when *p* < 0.05.

## Results

A total of 784 participants, 612 without DR (male, 66.34%) and 172 with DR (male, 65.70%), were included in the final sample analysis. The clinical characteristics of the two groups are presented in Table 1. Compared with non-DR participants, the DR diagnostic group tended to be older, higher SBP and a longer diabetes duration. In terms of glycemic indicators, subjects with DR had significantly higher FPG (8.62 ± 2.90 vs. 7.77 ± 2.33; *p* < 0.001) and 2hPG (15.19 ± 4.37 vs. 13.91 ± 4.08; *p* = 0.001), as well as higher HbA1c (9.21 ± 1.78 vs. 8.33 ± 2.15; *p* < 0.001). Additionally, participants with DR had significantly higher CRP (4.09 ± 1.47 vs. 2.96 ± 1.09; *p* < 0.001) and CRP/HDL-C (4.03 ± 1.67 vs. 2.66 ± 0.97; *p* < 0.001). Whereas, the HDL-C level and hemoglobin concentration were lower in the DR group. There was no significant difference in other indexes (*p* > 0.05).

**Table 1:** Characteristics of participants with T2DM stratified by without or with DR.

Variables	Non-DR group (*n* = 612)	DR group (*n* = 172)	*t/Z/χ* ^2^	*p*-value
Age (year)	62.15 ± 8.97	65.07 ± 8.07	−3.851	<0.001
Male (*n*, %)	406, 66.34%	113, 65.70%	0.025	0.927
Smoking (*n*, %)	121, 19.77%	51, 29.65%	0.052	0.852
Drinking (*n*, %)	145, 23.69%	27, 15.70%	1.244	0.318
SBP (mmHg)	133.27 ± 17.34	139.81 ± 16.34	−4.407	<0.001
DBP (mmHg)	80.78 ± 11.48	81.84 ± 10.29	−1.096	0.274
Duration (year)	8.55 ± 6.14	12.00 ± 5.69	−6.575	<0.001
FPG (mmol/L)	7.77 ± 2.33	8.62 ± 2.90	−3.552	<0.001
2 h-PG (mmol/L)	13.91 ± 4.08	15.19 ± 4.37	−3.248	0.001
FCP (pmol/L)	656.56 ± 308.90	611.74 ± 338.94	1.558	0.120
HbA1c (%)	8.33 ± 2.15	9.21 ± 1.78	−5.350	<0.001
AST (U/L)	22.42 ± 16.60	20.16 ± 14.25	1.623	0.105
ALT (U/L)	27.53 ± 24.84	23.51 ± 23.40	1.899	0.058
BUN (mmol/l)	6.70 ± 3.28	6.80 ± 1.68	−0.101	0.920
Cr (umol/l)	62.49 ± 20.87	64.82 ± 23.48	−1.178	0.240
UA (umol/l)	341.30 ± 86.97	340.91 ± 90.21	0.052	0.959
TG (mmol/l)	1.60 ± 1.11	1.81 ± 1.33	−1.911	0.057
TC (mmol/l)	4.33 ± 1.01	4.30 ± 1.14	0.303	0.762
HDL-C (mmol/l)	1.15 ± 0.27	1.07 ± 0.27	3.375	<0.001
LDL-C (mmol/l)	2.53 ± 0.83	2.49 ± 0.94	0.497	0.619
CRP (mg/l)	2.96 ± 1.09	4.09 ± 1.47	−9.378	<0.001
HBG (g/l)	143.66 ± 14.86	140.18 ± 15.81	2.657	0.008
CRP/HDL-C	2.66 ± 0.97	4.03 ± 1.67	−10.304	<0.001

DR, diabetic retinopathy; SBP, systolic blood pressure; DBP, diastolic blood pressure; FPG, fasting plasma glucose; 2hPG, 2-h postprandial blood glucose; FCP, fasting plasma C-peptide; HbA1c, glycated hemoglobin A; AST, aspartate aminotransferase; ALT, alanine aminotransferase; BUN, blood urea nitrogen; Cr, creatinine; UA, uric acid; TG, triglycerides; TC, total cholesterol; HDL-C, high-density lipoprotein cholesterol; LDL-C, low-density lipoprotein cholesterol; CRP, C-reactive protein; HBG, hemoglobin.

Based on the quartiles of the CRP/HDL-C ratio at baseline, the patients were divided into four groups. Specifically, Quartile 1 (Q1): CRP/HDL-C < 2.087, *n* = 196; Quartile 2 (Q2): CRP/HDL-C 2.088 ~ 2.764, *n* = 196; Quartile 3 (Q3): CRP/HDL-C 2.765 ~ 3.551, *n* = 196; Quartile 4 (Q4): CRP/HDL-C > 3.551, *n* = 196. The prevalence of DR across the Q1, Q2, Q3 and Q4 groups was7.65, 15.31, 19.90, and 44.90%, respectively, indicating a significant upward trend (P for trend<0.001). Then, taking Q1 as the reference group, Q2, Q3 and Q4 all showed a significant association with increased incidence of DR (Q2, OR 2.218, 95% CI: 1.133 ~ 4.197, *p* = 0.020; Q3, OR 2.997, 95% CI: 1.592 ~ 5.643, *p* < 0.001; Q4, OR 9.832, 95% CI: 5.412 ~ 17.864, *p* < 0.001) after the logistic regression analysis (Table 2). Further, after adjustment for age, SBP, diabetes duration, FPG, 2 h-PG, HbA1c, and HBG, compared to the Q1 of CRP/HDL-C, the Q3 and Q4 groups still had a markedly increased risk of DR by 190.5% (OR 2.905, 95% CI: 1.372 ~ 6.152, *p* = 0.005) and 893.8% (OR 9.938, 95% CI: 4.987 ~ 19.804, *p* < 0.001), separately, while no difference was observed in Q2 group (OR 1.803, 95% CI: 0.825 ~ 3.940, *p* = 0.139).

**Table 2:** Results of logistic regression analysis comparing the effects of different CRP/HDL-C level subgroups on DR.

CRP/HDL-C Quartile	*n* (Total/DR)	Model 1			Model 2		
		*β*	OR (95% CI)	*p*-value	*β*	OR (95% CI)	*p*-value
Q1 (<2.087)	196/15	–	1.00 (Reference)	–	–	1.00 (Reference)	–
Q2 (2.088 ~ 2.764)	196/30	0.780	2.218 (1.133 ~ 4.197)	0.020	0.589	1.803 (0.825 ~ 3.940)	0.139
Q3 (2.765 ~ 3.551)	196/39	1.098	2.997 (1.592 ~ 5.643)	<0.001	1.066	2.905 (1.372 ~ 6.152)	0.005
Q4 (>3.551)	196/88	2.286	9.832 (5.412 ~ 17.864)	<0.001	2.347	9.938 (4.987 ~ 19.804)	<0.001

DR, diabetic retinopathy; HDL-C, high-density lipoprotein cholesterol; CRP, C-reactive protein; OR, odd ratio; CI, confidence interval.

Model 1: Unadjusted model.

Model 2: Adjusted for Age, SBP, Duration, FPG, 2 h-PG, HbA1c, and HBG.

**Figure 1:**
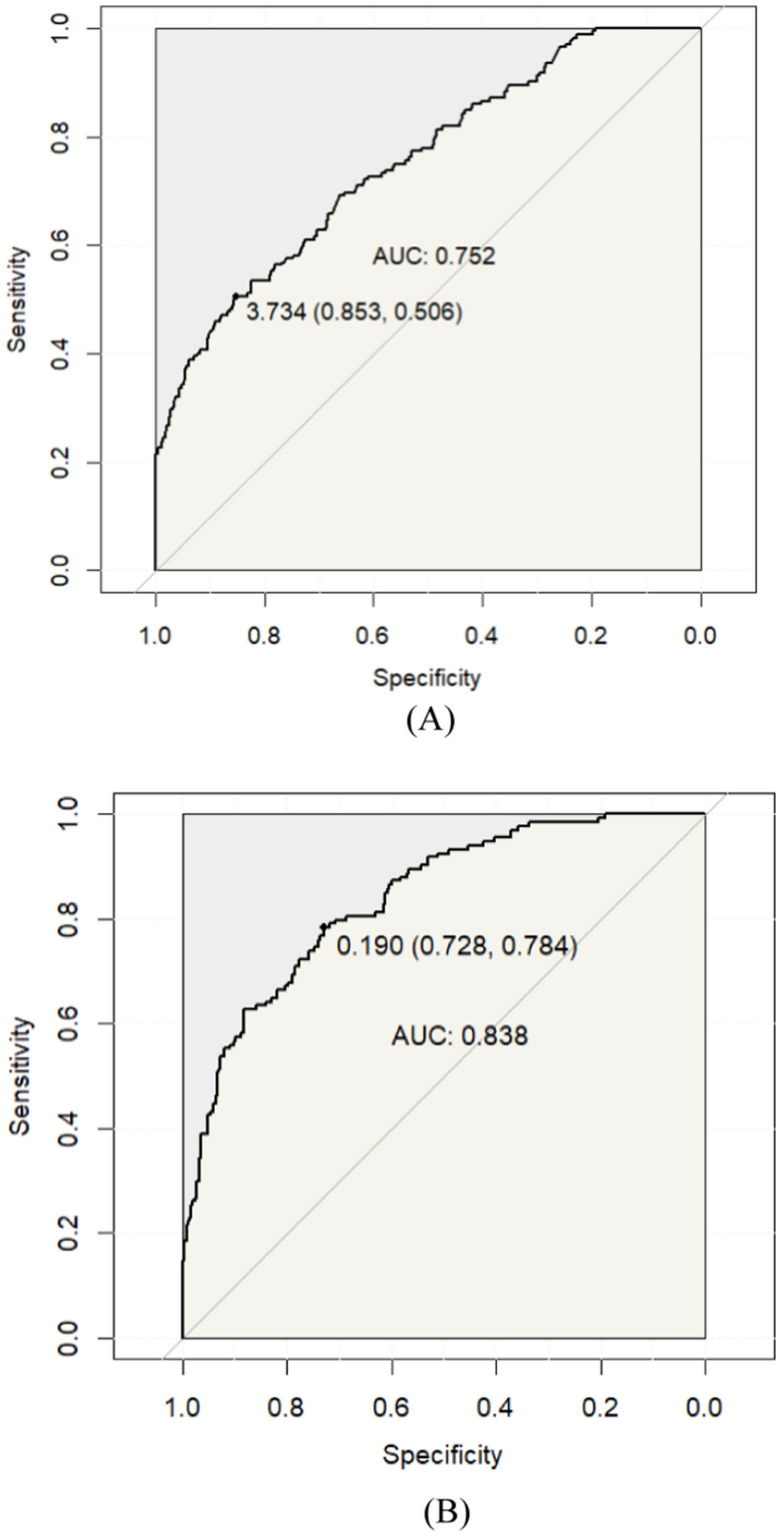
Receiver operating characteristic curves (ROC) of DR in T2DM. **(A)** CRP/HDL-C; **(B)** CRP/HDL-C combined other 4-indicators (Age, Duration, FPG, and HbA1c).

Furthermore, to investigate which indexes were vulnerable to increased DR occurrence, the relationship of DR occurrence with different indexes was evaluated. Based on the findings of univariate analysis, the following parameters were entered in the multivariate logistic regression model: age, SBP, diabetes duration, FPG, 2 h-PG, HbA1c, HDL-C, CRP, HBG, CRP/HDL-C. After conducted, the odds ratios (ORs) of DR showed that age (OR 1.036, 95% CI: 1.004 ~ 1.069, *p* = 0.029), diabetes duration (OR 1.106, 95% CI: 1.061 ~ 1.153, *p* = 0.001), FPG (OR 1.126, 95% CI: 1.003 ~ 1.264, *p* = 0.045), HbA1c (OR 1.197, 95% CI: 1.051 ~ 1.363, *p* = 0.007) and the CRP/HDL-C ratio (OR 3.176, 95% CI: 1.280 ~ 7.877, *p* = 0.013) were identified as risk factors for DR (Table 3). Moreover, we conducted the receiver operating characteristic (ROC) curve analysis and area under the curve (AUC) to evaluate the predictive value of parameters for the risk of DR occurrence. The CRP/HDL-C ratio had the best diagnostic performance AUC = 0.752 (95% CI: 0.711 ~ 0.794), followed by diabetes duration AUC = 0.672(95% CI: 0.626 ~ 0.719), HbA1c AUC = 0.653 (95% CI: 0.605 ~ 0.701), FPG AUC = 0.636 (95% CI: 0.580 ~ 0.692) and age AUC = 0.557 (95% CI: 0.500 ~ 0.613). Then, we combined indicators of the five AUCs and found that the AUC of the mixture could reach 0.838 (95% CI: 0.801 ~ 0.875) (Figure 1).

**Table 3:** Logistic regression analysis of risk factors for DR in T2DM patients.

Variables	Univariable regression				Multivariable regression			
	*β*	SE	OR (95% CI)	*p*-value	*β*	SE	OR (95% CI)	*p*-value
Age (year)	0.042	0.011	1.043 (1.021 ~ 1.066)	<0.001	0.035	0.016	1.036 (1.004,1.069)	0.029
SBP (mmHg)	0.022	0.005	1.022 (1.012 ~ 1.033)	<0.001				
Duration (year)	0.089	0.014	1.093 (1.063 ~ 1.124)	<0.001	0.1010	0.021	1.106 (1.061 ~ 1.153)	0.001
FPG (mmol/L)	0.129	0.033	1.137 (1.066 ~ 1.214)	<0.001	0.119	0.059	1.126 (1.003 ~ 1.264)	0.045
2 h-PG (mmol/L)	0.073	0.023	1.075 (1.029 ~ 1.124)	0.001				
HbA1c (%)	0.190	0.041	1.210 (1.116 ~ 1.311)	<0.001	0.180	0.066	1.197 (1.051 ~ 1.363)	0.007
HDL-C (mmol/l)	−1.140	0.343	0.320 (0.163 ~ 0.626)	<0.001				0.799
CRP (mg/l)	0.700	0.076	2.013 (1.736 ~ 2.335)	<0.001				0.619
HBG (g/l)	−0.015	0.006	0.985 (0.974 ~ 0.996)	0.008				0.244
CRP/HDL-C	0.885	0.087	2.423 (2.044 ~ 2.872)	<0.001	1.156	0.464	3.176 (1.280 ~ 7.877)	0.013

DR, diabetic retinopathy; SBP, systolic blood pressure; FPG, fasting plasma glucose; 2 h-PG, 2-h postprandial blood glucose; HbA1c, glycated hemoglobin A; HDL-C, high-density lipoprotein cholesterol; CRP, C-reactive protein; HBG, hemoglobin; OR, odd ratio; CI, confidence interval.

## Discussion

In the current study, we found that after adjustment for the impact of various covariates, the odds ratio of the third and fourth vs. the first quartile of CRP/HDL-C were 2.905 (95% CI: 1.372 ~ 6.152) and 9.938 (95% CI: 4.987 ~ 19.804), respectively, while no significant correlation was observed in the second vs. the first quartile of CRP/HDL-C. Additionally, the CRP/HDL-C ratio is independently and positively associated with the occurrence of DR in patients with T2DM (OR 3.176, 95% CI: 1.280 ~ 7.877; *p* = 0.013), which highlighted CRP/HDL-C level raise as a high-risk factor for higher risk of DR. It is implied that the CRP/HDL-C ratio can be applied as a risk predictor for DR in T2DM. In addition, we confirmed that age, FPG, HbA1c and diabetes duration are strongly associated with DR risk.

Diabetic retinopathy (DR) as one of the most common microvascular complications of DM (16), remains a leading cause of preventable blindness in the adult working population and the global DR burden is expected to remain high through 2045 (4). Approximately 19.5 million people have diagnosed with DR, the overall prevalence of any DR among those aged 18–74 was 16.3% in our country (17). With the increasing prevalence of diabetes globally and increasing longevity and aging of the global population, DR has consequently become a serious global health challenge. Therefore, it is very important to find more predictors for DR.

The combination of CRP level and lipid indices, CRP/HDL-C, is applied in clinical practice as a predictor of metabolic syndrome (12, 13). Compared with a single indicator, it can simultaneously consider the correlation between lipid metabolism, inflammatory metabolism and DR in T2DM patients, thereby improving the accuracy of clinical prognosis prediction. According to the present study, there is a correlation between the CRP/HDL-C ratio and the likelihood of DR risk. Lipid metabolism and inflammation have been reported to be among the most affected pathways in the proteome or metabolome of various tissues affected by diabetes (18). Dyslipidemia is a pivotal pathological mechanism in DM and its complications. Lipid levels depend on a variety of regulatory mechanisms, among which the regulation of reverse cholesterol transport and its specific receptors, has been receiving increasing attention in relation to inflammation, especially DR and diabetic nephropathy (19, 20). Moreover, previous studies have demonstrated that inflammatory processes play an essential role in the pathogenesis of DR, increasing retinal vascular permeability and neovascularization, as well as leading to retinal neurodegeneration, which in turn accelerates DR progression (21). The interplay between lipid metabolism and inflammation is of utmost importance in the development of DR. Several studies reported that plasma HDL-C elevation has been considered a therapeutic approach to reduce the risk of T2DM and the vascular complications associated with DM (22, 23). The plasma levels of CRP exhibit a positive correlation with the severity of inflammation, making it a commonly employed nonspecific biomarker in clinical practice (24). Meanwhile, CRP is the most widely studied of the inflammatory markers associated with cardiovascular diseases (CVDs), and has even been used as the “gold standard” for CVD risk assessment. A prospective cohort study found that CRP as a mediator biomarker mediated a higher risk of microvascular complications in patients with T2DM (25). Qiu et al. reported that human CRP-induced overexpression of pro-inflammatory, pro-oxidative, and pro-angiogenic factors was associated with up-regulation of CD32 and the NF-κB signaling in the retinas, suggesting that elevated CRP levels play a pathogenic role in DR (26). Therefore, the CRP/HDL-C ratio could provide a more accurate and comprehensive reflection of the severity of DR risk in patients with T2DM. Similarly, our study confirmed the CRP/HDL-C ratio good diagnostic value in assessing the risk of DR occurrence with an area under the ROC curve of 0.752, also suggested that elevated CRP/HDL-C levels are connected with a higher risk of DR, and changes of this composite indicator should be emphasized in clinical care, especially in T2DM patients with CRP/HDL-C higher than 3.734.

This study has a number of benefits. First, to our knowledge, this study is the first to report the relationship between the CRP/HDL-C ratio and the risk of diabetic retinopathy in T2DM patients, which provides a new sight for predicting the incidence of retinopathy. Second, this study had a relatively large sample size and sufficient clinical information, and the associations of the CRP/HDL-C ratio and risk of DR remained stable even after multivariable adjustments, making the produced results more compelling. Although our study provided CRP/HDL-C, as an inflammation-lipid composite marker, can predict of DR by the simple and easy calculation, some limitations should be acknowledged. First, the study was a single-center cross-sectional study, and there is a disproportion in number of cases with and without retinopathy, in addition, we did not compare the CRP/HDL-C ratio with other known biomarkers (e.g., PTX3) enables a more comprehensive assessment of its predictive value, so it is indispensable to future validate by expanding the sample size and including multicenter data to improve the generalizability and reliability of our findings. Second, our study did not demonstrate a direct causal relationship between CRP/HDL-C and DR. Third, although we conducted rigorous adjusting for potential confounders, the possibility of residual confounders cannot be ruled out, such as the treatment of cardiovascular medications.

## Conclusion

Our study elucidated the association between the risk of diabetic retinopathy (DR) and the CRP/HDL-C ratio in patients with T2DM, the higher CRP/HDL-C levels were correlated with the higher risk of DR. The CRP/HDL-C ratio is a significant predictor of DR. A composite index based on the CRP/HDL-C ratio effectively predicts the risk of DR.

## Data Availability

The raw data supporting the conclusions of this article will be made available by the authors, without undue reservation.
